# The role of *ATP1A3* gene in epilepsy: We need to know more

**DOI:** 10.3389/fncel.2023.1143956

**Published:** 2023-02-13

**Authors:** Shuang Zou, Yu-Long Lan, Yiwei Gong, Zhong Chen, Cenglin Xu

**Affiliations:** ^1^Key Laboratory of Neuropharmacology and Translational Medicine of Zhejiang Province, School of Pharmaceutical Science, Zhejiang Chinese Medical University, Hangzhou, China; ^2^Department of Neurosurgery, Second Affiliated Hospital, School of Medicine, Zhejiang University, Hangzhou, Zhejiang, China

**Keywords:** ATP1A3, epilepsy, Na^+^/K^+^-ATPase, animal models, mutation, mechanism, treatments ATP1A3, treatments

## Abstract

The *ATP1A3* gene, which encodes the Na^+^/K^+^-ATPase α3 catalytic subunit, plays a crucial role in both physiological and pathological conditions in the brain, and mutations in this gene have been associated with a wide variety of neurological diseases by impacting the whole infant development stages. Cumulative clinical evidence suggests that some severe epileptic syndromes have been linked to mutations in *ATP1A3*, among which inactivating mutation of *ATP1A3* has been intriguingly found to be a candidate pathogenesis for complex partial and generalized seizures, proposing *ATP1A3* regulators as putative targets for the rational design of antiepileptic therapies. In this review, we introduced the physiological function of *ATP1A3* and summarized the findings about *ATP1A3* in epileptic conditions from both clinical and laboratory aspects at first. Then, some possible mechanisms of how *ATP1A3* mutations result in epilepsy are provided. We think this review timely introduces the potential contribution of *ATP1A3* mutations in both the genesis and progression of epilepsy. Taken that both the detailed mechanisms and therapeutic significance of *ATP1A3* for epilepsy are not yet fully illustrated, we think that both in-depth mechanisms investigations and systematic intervention experiments targeting *ATP1A3* are needed, and by doing so, perhaps a new light can be shed on treating *ATP1A3*-associated epilepsy.

## 1. Introduction

The electrogenic activity of sodium (Na^+^)/potassium (K^+^)-ATPase, an ionic pump driven by ATP hydrolysis, is essential for normal neuronal physiology, homeostasis, and signaling by maintaining Na^+^ and K^+^ gradients across the plasma membrane. The basic structure of Na^+^/K^+^-ATPase has been previously elucidated; it contains one large catalytic subunit (α) and two small auxiliary subunits (β, FXYD), which are composed of a Na^+^/K^+^-ATPase complex (Holm et al., 2016). The enriched α-subunits in vertebrate brains are encoded by paralogous genes (*ATP1A1* to *3*) that share a common gene promoter (McGrail et al., 1991). Among these genes, *ATP1A3* encodes the Na^+^/K^+^-ATPase α3 isozyme, which is mainly expressed in the brain and highly expressed in neurons, and serves as the catalytic component of the pump because it contains binding sites for Na^+^, K^+^, ATP, and cardiac glycosides (pump inhibitors). The *ATP1A3* isoform is particularly crucial for restoring electrochemical gradients in neuronal activities (Laird et al., 2015; Holm et al., 2016) since *ATP1A3* is expressed exclusively in neurons, while others are not (McGrail et al., 1991).

Despite physiological conditions, substantial evidence has suggested that pathogenic variants in *ATP1A3* are potential causes of various neurological disorders (Smedemark-Margulies et al., 2016; Salles et al., 2021; Vezyroglou et al., 2022). Interestingly, the disease onsets seem to extensively span among all ages, even though those disorders are associated with genetic mutations in *ATP1A3*, suggesting that dysfunction of vulnerable *ATP1A3* in various forms could occur in childhood as the brain develops, but its impact may also emerge in the later adulthood. Single-cell exploration has pointed toward a critical role for *ATP1A3* in human brain development and a cell type basis for *ATP1A3*-associated neurological disorders (Smith et al., 2021). Multiple studies have already shown the causal significance of *ATP1A3* polymorphisms in developing various neurological diseases with a common inheritance mode (Heinzen et al., 2014; Rosewich et al., 2017; Carecchio et al., 2018; Capuano et al., 2020). Here, we mainly focus on the specific functions of *ATP1A3* in epilepsy which is a common and intractable chronic neurological disease. Delighted by related findings from both clinical and laboratory aspects, in this review, the alterations of *ATP1A3* in epilepsy were initially introduced, followed by some related mechanisms. Then, we raised some prospections in translational therapeutic views for both controlling seizures and interfering with epileptogenesis caused by *ATP1A3* mutations.

## 2. Clinical evidence about *ATP1A3* alterations in epilepsy

Currently, various studies have demonstrated that 0.3% of the entire population is affected by genetic generalized epilepsies (GGEs), nearly accounting for 30% of all the patients who suffer from epilepsies (Reid et al., 2009). Genetic generalized epilepsies are found to be polygenic and primarily include juvenile myoclonic epilepsy (JME) or epilepsy with generalized tonic-clonic seizures (EGTCS) alone (Weber and Lerche, 2008). All of these share a high incidence of pharmacoresistance, and the management of intractable pharmacoresistant epilepsy emerges as a great burden for those patients with epilepsy (PWE) (Xu et al., 2022). Thus, revealing the exact genetic mutations and related pathogenesis factors of genetic epilepsy would largely benefit those patients for both prognosis and management purposes.

### 2.1. ATP1A3 mutation is related to a higher risk of developing epilepsy

It must be admitted that most previous efforts have focused on the mutations in genes encoding ion channels or synaptic receptors which directly cause abnormal hyperexcitability in the brain (Hanada, 2020). The existence of *ATP1A3* mutations in epilepsy seems to be the “elephant in the room”. Although somehow limited direct data have been presented, various clinical evidence could still support the essential roles of *ATP1A3* in the incidence of epilepsy. Until now, more than 243 mutations (missense, 214; fusion, 1; nonsense, 8; splice, 14; FS del, 4; FS ins, 2) in *ATP1A3* have been reported since the initial reports appeared in 2003 and 2004 (De Fusco et al., 2003; De Carvalho Aguiar et al., 2004) (cBioPortal data, http://www.cbioportal.org, last accessed January 2023). Among these, various mutations could be of great potential in inducing early-onset epilepsy (Ishii et al., 2013; Paciorkowski et al., 2015; Marzin et al., 2018; Schirinzi et al., 2018; Younes et al., 2018; Ishihara et al., 2019) (Table 1). Some researchers have proposed a phenotypic continuum for the autosomal dominant diseases caused by *ATP1A3* mutations, encompassing alternating hemiplegia of children (AHC), rapid-onset dystonia-parkinsonism (RDP), relapsing encephalopathy with cerebellar ataxia (RECA), cerebellar ataxia, areflexia, pes cavus, optic atrophy and sensorineural hearing loss (CAPOS), and developmental and epileptic encephalopathies (DEE) (Rosewich et al., 2014; Dard et al., 2015; Paciorkowski et al., 2015).

**Table 1:** Summary of clinical findings involving ATP1A3-related epilepsy.

**Authors**	**Year**	**ATP1A3 mutation**	**Patient number**	**Key findings/objectives**
Ishii et al.	2013	c.2443G>A (p.E815K)	5	The authors reported that the E815K mutation of ATP1A3 found in half of their patients was associated with the presence of severe neurological symptoms, status epilepticus and resistance to medications.
Paciorkowski et al.	2015	c.1073G>T (p.G358V)	2	Such mutation could cause severe phenotypes of ATP1A3-related disorder spectrum that include catastrophic early life epilepsy.
Schirinzi et al.	2018	c.2266_2268delGAC (p.D756del)	1	The authors described a patient who developed a severe early onset drug-resistant epileptic encephalopathy, and interestingly a novel mutation in ATP1A3 gene was found in this case.
Marzin et al.	2018	c.2224G>T (p.D742Y)	1	The patient had an unreported heterozygous de novo sequence variant in ATP1A3, and have been found to be of a phenotype characterized by early-onset attacks of movement disorders, which proved to be epileptic, and severe developmental delay.
Marzin et al.	2018	c.1036 T>C (p.C346R)	1	The same as above.
Marzin et al.	2018	c.1825G>T (p.D609Y)	1	The same as above.
Younes et al.	2018	c.1111G> A (p.G371S)	1	The authors reported on early life epilepsy with episodic apnea potentially secondary to ATP1A3 mutation in a Tunisian child.
Ishihara et al.	2019	c.2736_2738CTTdel (p.F913del)	1	The authors reported a neonatal case of catastrophic early life epilepsy, associated with a novel heterozygous mutation in the ATP1A3 gene, a single amino acid deletion in the 8th transmembrane domain of ATP1a3 (F913del).

Multiple autosomal dominant neurological diseases with limited overlap have been linked to *ATP1A3* mutations in constitutional heterozygotes (Dard et al., 2015). Previous research has linked *ATP1A3* mutations to the most severe form of infantile epileptic encephalopathy, characterized by seizures beginning at infancy, episodic apnea, poor survival, and profound developmental retardation (Sasaki et al., 2014; Paciorkowski et al., 2015). Patients with RDP are less likely to have epileptic seizures than those with AHC in which more than half of individuals would experience seizures (Bøttger et al., 2012). Patients with clinically diagnosed AHC have been found to suffer untreatable newborn seizures, status epilepticus, and early-life epilepsy (Saito et al., 2010; Ishii et al., 2013; Rosewich et al., 2014). To ascertain whether these frequent variations play significant roles in the susceptibility to generalized genetic epilepsies, Qu et al. (2015) screened 484 patients with GGE and 284 healthy controls for eight tagSNPs in *ATP1A2* and *ATP1A3*. According to the authors' hypothesis, frequent variations of *ATP1A3* increase one's risk of developing GGEs. Evidences have been found linking a common variation of *ATP1A3* (*ATP1A3* rs8107107) to a higher risk of developing epilepsy, and in particular, epilepsy with generalized tonic-clonic seizures. These results proved that the *ATP1A3* gene is involved in the etiology of generalized genetic epilepsies. The following studies further demonstrated that severe epilepsies have been reported in people with *ATP1A3* mutations, and intellectual impairment may concomitantly occur with FHM and AHC (Paciorkowski et al., 2015; Liu et al., 2018).

In addition to those PWEs whose epilepsy was directly caused by the mutation in the *ATP1A3* isoform, patients with other *ATP1A3*-associated neurological diseases are also reported to be at high risk for concomitant seizures. An early study published in 2015 estimated that more than 50–80% of all alternating hemiplegia of childhood (AHC) cases in the literature had a history of seizures (Paciorkowski et al., 2015), while the incidence rate is lower (26.1%) in a later study (Li et al., 2018). This difference may be attributed to the different sample sizes; however, the incidence rate of epilepsy was significantly higher than in normal populations (nearly 1%). Despite the heterogeneity of *ATP1A3*-related seizure types, specific patterns have emerged. Seizures begin in childhood and range from the focal or generalized tonic, tonic-clonic to myoclonic attacks and are triggered by secondary external factors, such as stress, excitement, temperature extremes, water exposure, physical effort, and changes in lighting (Younes et al., 2018).

Thus, generally speaking, as a mutational susceptible gene, *ATP1A3* alteration could easily lead to developmental epilepsy, normally characterized by more frequent seizures, more serious symptoms as well as poor treatment efficacy. While the decreased activity of *ATP1A3* may participate, current existing evidence is still not sufficient to totally explain the observed clinical phenomenon. When *ATP1A3* is mutated, it causes neurological abnormalities that range widely in symptomatology and severity. This might be the reason why *ATP1A3*-related epilepsy usually shows a worse prognosis. Interestingly, D923N, related to hypotonia or dystonia with an average onset age of 3 years, and L924P, which is associated with severe infantile epilepsy and profound disability, have already been examined (Arystarkhova et al., 2021). In this study, the authors showed that the eIF2α inhibition, ER retention, lactacystin response, and membrane redistribution on sucrose density gradients were all affected differently in humans with these two *ATP1A3* mutations. As the L924P mutation has a more severe phenotype, misfolding during biosynthesis likely contributes to pathogenicity in addition to the enzyme's loss of activity (Einholm et al., 2010; Holm et al., 2016). Based on the clinical evidence, we have already recognized the potential roles of *ATP1A3* in the incidence of epilepsy clinically. However, the critical roles of *ATP1A3* still need further validation on more and larger population groups, especially focusing on the clear elaboration of *ATP1A3* mutation-related family gene expression pattern.

### 2.2. Diversities in mutations characteristics of ATP1A3 in epilepsy

Although decreased activity may play a role, a molecular foundation for the epilepsy-promoting effects of *ATP1A3* mutations is yet unknown. Most mutations had no clear relationship between the mutation site and phenotype (Arystarkhova et al., 2019). The protein misfolding and pump deactivation are thought to have unique biological implications (Arystarkhova et al., 2019). The authors showed that whereas a mutation that affects Na^+^/K^+^-ATPase function would cause moderate illness, one that triggers the unfolded protein response might cause severe disease and, perhaps, the death of neurons even if the ion pump is not disabled.

**Figure 1:**
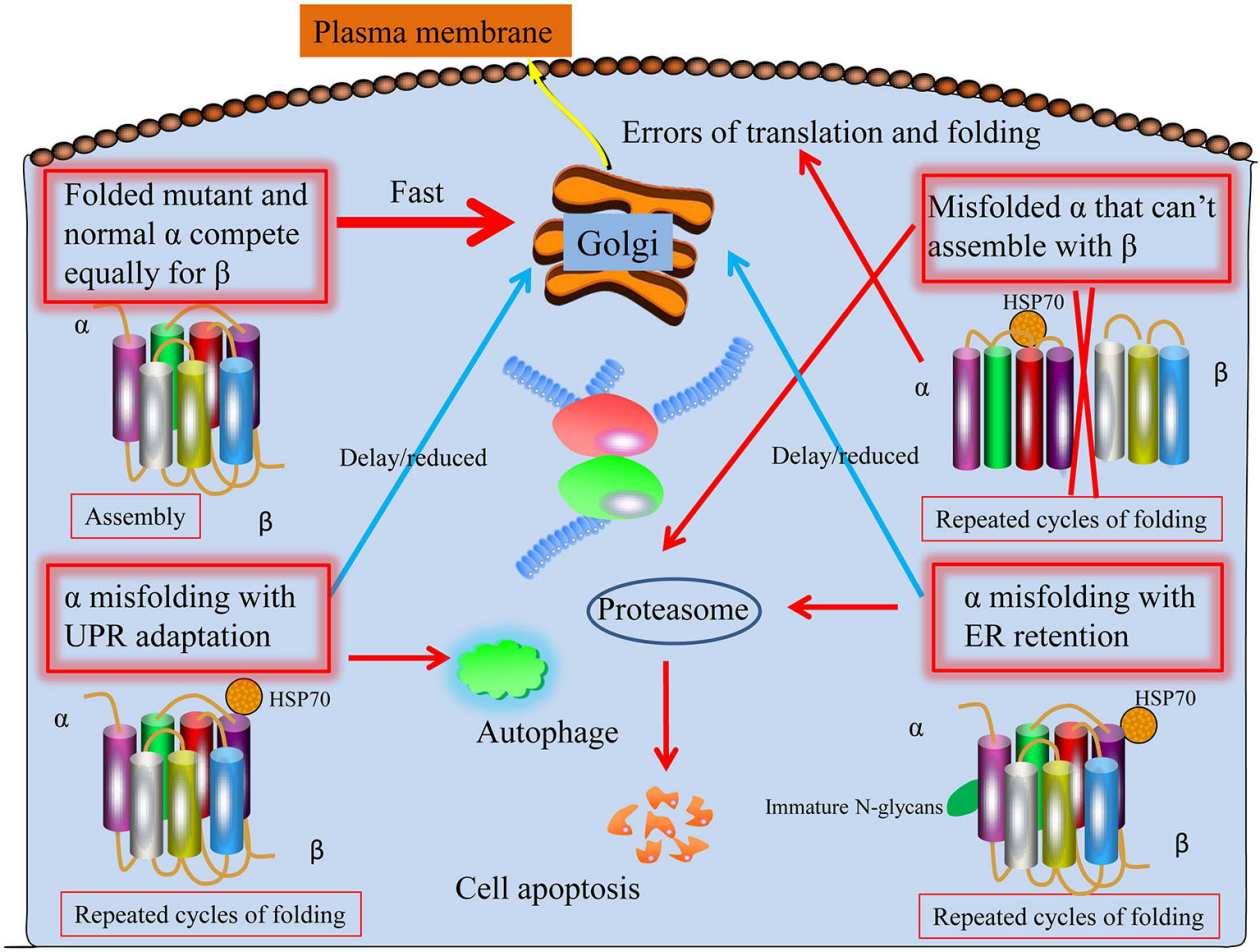
Proposed four potential ways ATP1A3 mutations may proceed through the biosynthetic process. First, the protein folds and biosynthesizes normally; second, it undergoes irreversible misfolding and is subsequently degraded by proteasomes (ERAD); third, the presence of a chaperone assists biosynthesis; and fourth, the chaperone assistance is ineffective. As with HSP70, the shown chaperone is meant to stand in for various chaperone classes. Mutant and normal ATP1A3 alleles compete for the β subunit, making it difficult to anticipate the effects of ATP1A3 mutations. It is believed that every particular mutation that might worsen epilepsy disease severity may lie among these four extremes.

Dominant *ATP1A3* mutations may cause a broad spectrum of neurological diseases. Predictions regarding the factors in the epilepsy disease severity of *ATP1A3* mutations can be made, and at least four ways things may go wrong (Arystarkhova et al., 2019). To start, a mutant protein with similar folding properties to the wild-type allele should be transported to the membrane (Figure 1). That is the worst-case scenario if a mutation has a gain-of-toxic function, such as ion leakage, but not if it is inactive. Second, a mutation that prevents the C-terminal third of the protein from folding (the obligatory β binding sites location) may have less of an effect: the α from the mutant allele will not compete with the normal allele if it is unable to interact with β. Similar to what has been described for several Drosophila Na^+^/K^+^-ATPase mutations, this might result in more β being accessible from the good allele to the developing α chains, leading to an improved outcome beyond that of haploinsufficiency (Ashmore et al., 2009). The unfolded mutant α will be extruded from the ER into the cytoplasm with the help of chaperones like BiP, where it will be ubiquitinated and degraded by the proteasome endoplasmic reticulum-associated degradation (ERAD), a mechanism that is always active to cope with the normal background of translation and folding mistakes. Third, even if faulty, misfolded proteins may try to traffic to the Golgi and beyond by engaging in calnexin- or calreticulin-mediated refolding cycles. The α subunit lacks the N-glycans necessary to associate with these chaperones. The unfolded protein response (UPR) involves the adaptive expansion of the ER and upregulation of proteins that promote folding in response to an increasing load of misfolded protein (Karagöz et al., 2019). This may modify the outcome of mutations that result in a gain-of-toxic function. Misfolded protein aggregates will trigger the UPR defensive reaction autophagy. Finally, the UPR will cause apoptosis, leading to the death of neuronal cells, if ER enlargement and autophagy are inadequate (Figure 1).

It has now been established that protein-modifying genetic variations in *ATP1A3* are uncommon in the general population and that when they do exist, they carry a very high risk of causing severe neurological disorders (Heinzen et al., 2014). Consequently, researchers have begun looking into the possibility that mutations in *ATP1A3* cause other disorders. If they exist, the potential involvement of ATP1A3 mutations in a broader spectrum of phenotypes may likely become evident as NGS becomes more frequently employed in routine clinical practice. More research is necessary to fully understand the vast spectrum of illness severity reported with *ATP1A3* mutations.

### 2.3. ATP1A3 mutation results in poor responses to anti-epileptic drugs (AEDs)

Anti-epileptic drugs (AEDs) are routinely used to control seizures in PWEs. However, one-third of the PWEs cannot achieve desirable outcomes, which is diagnosed as pharmacoresistant epilepsy (Kwan and Brodie, 2000). Pharmacoresistant epilepsy usually shares more severe pathological conditions including hyperexcitable circuitry and abnormally activated molecular pathways (Xu et al., 2019, 2021), resulting in the intractability of successful management. With increased clinical findings, it is somehow convincible that epilepsy with *ATP1A3* mutation shares a high incidence of being pharmacoresistant. Gasser et al. (2020) analyzed the frequency of their usage and the effectiveness of these treatments in individuals with *ATP1A3*-related seizures. Although the therapeutic responses to AEDs are seldom recorded in individuals with *ATP1A3*-related seizures. As a result, very few patients were included in their study based on their systematic reports and patient data. However, among the total 21 patients, only 12 (57%) reported a reduction in both the frequency and severity of seizures. In 43% of the patients, no substantial response to seizure medication was observed, which is far higher than normal PWEs (30% of which would become pharmacoresistant). According to Schirinzi et al., in a patient with severe early-onset drug-resistant epileptic encephalopathy, another new mutation in the *ATP1A3* gene was also discovered (Schirinzi et al., 2018), supporting the hypothesis that *ATP1A3* mutation would induce poor responses to AEDs. The answers to why *ATP1A3* mutations resulted in poor responses to current AEDs may be attributed to that when the *ATP1A3* mutations are placed in their proper context, a possible chain of events may be envisioned: some people are predisposed to status epilepticus (SE) because of pathogenic *ATP1A3* gene variants; SE may increase the risk of recurrent seizures; and damage the mesial temporal lobe in a way that reduces GABA-mediated inhibition or misdirects regeneration, leading to a novel recurrent excitatory circuit (Sills, 2007). Also, our very recent research has already confirmed that Na^+^/K^+^-ATPase emerges as a potential anti-seizure target (Zhao et al., 2022). It is plausible to speculate that dysfunction of the *ATP1A3* gene, which is a crucial part of Na^+^/K^+^-ATPase, would lead to a more seizure-susceptible and hyper-excitable brain that would directly lead to pharmacoresistance (Xu et al., 2019). However, the precise mechanisms regarding the roles of *ATP1A3* in the drug sensitivity of epilepsy still need more evidence.

Intriguingly, epileptic seizures of an *ATP1A3* mutated PWE were controlled when the ketogenic diet (KD) was started (Schirinzi et al., 2018). These findings further proposed the use of KD, which has become more and more popular as a treatment for drug-resistant epilepsy and other neurological diseases, as an innovative treatment for *ATP1A3*-related diseases. Although definitive confirmatory trials of KD are still required. The anti-epileptic effect of KD has been reported to be related to several factors, including direct anti-seizure action, the restoration of a new neurotransmitter balance, alterations in mitochondrial and cellular metabolism, and an antioxidant influence (Rho, 2017). However, the unique activity of ketones on ATP-sensitive potassium channels (KATP channels) may be responsible for the success of KD in the above situation. KATP channels (activity altered by *ATP1A3* malfunction) are thought to be responsible for the beneficial effects of ketosis on epilepsy (ref), which makes sense that dysfunction of the Na^+^/K^+^-ATPase caused by *ATP1A3* mutation, which induces hyperexcitability in neurons, would also occasionally lead to inhibition of KATP channels (Tanner et al., 2011). Additionally, KD therapy may reverse this condition to reduce neuronal excitability. Their patient's clinical path and the success of KD imply the possibility of using this approach to treat *ATP1A3*-related epilepsy or other disorders. However, more systematic clinical trials with enough sample sizes are urgently needed.

However, most current papers on pharmaco-resistant epilepsy associated with *ATP1A3* mutations did not provide any tips on how to manage seizures. The current treatment experiences are often ambiguous and inadequate for properly assessing the efficacy of AEDs. Consequently, further research is required to first identify the specific types of AEDs associated with *ATP1A3* mutations and to seek a more prominent combination of multiple treatment strategies in the clinical arena.

## 3. Laboratory efforts addressing the role of *ATP1A3* in epileptogenesis and epilepsy

Besides clinical investigations, animal studies provide great platforms for both intervention convenience and in-depth mechanism insights for addressing the role of *ATP1A3* as well as Na^+^-K^+^-ATPase in epilepsy from the bench side.

### 3.1. Evidence about ATP1A3 in experimental epilepsy and possible mechanisms

Currently, increasing numbers of laboratory studies have unveiled the critical roles of Na^+^-K^+^-ATPase in epilepsy (Ygberg et al., 2021). In rodent models of epilepsy, the activity of Na^+^-K^+^-ATPase has been reported to be altered, and pharmacological inhibition of Na^+^-K^+^-ATPase would cause epileptic seizures in rodents (Holm and Lykke-Hartmann, 2016). Mechanically, Na^+^-K^+^-ATPase inhibition could lead to intracellular Na^+^ accumulation, which will further result in the increased NMDA receptor-mediated excitation and inhibition of inward transportation of GABA, as well as the accumulation of intercellular free Ca^2+^ through the reversion of intercellular Na^+^-Ca^2+^ exchanger (Sun et al., 2022). In addition, Na^+^-K^+^-ATPase dysfunction could also be related to a decrease in intracellular K^+^ homeostasis, resulting in impaired glutamate clearance and a depolarized change in resting membrane potential (Shao et al., 2021). Furthermore, as we recently reported, activation of astrocytic Na^+^-K^+^-ATPase would achieve great anti-seizure outcomes in neocortical epileptic mice (Zhao et al., 2022). With such substantial reported data, Na^+^-K^+^-ATPase is certainly a crucial contributor to brain excitability in epileptic brains (Sun et al., 2022). Importantly, the presence of epileptic symptoms in various neurological disorders caused by Na^+^-K^+^-ATPase subunits mutations suggested the causal relationship between Na^+^-K^+^-ATPase activity and epilepsy (Vezyroglou et al., 2022).

With regard to *ATP1A3*, clinical literature has demonstrated that, as a mutational susceptibility gene, *ATP1A3* alteration could easily lead to developmental epilepsy, which normally exhibits more frequent seizures, more serious symptoms, and poor AEDs responses. Besides clinicians, laboratory researchers also devote their efforts to investigating the role and mechanisms of *ATP1A3* in epileptic animals. The beginning of related studies can be traced back to the 2000s, and various early explorations have indicated the important roles of *ATP1A3* in epileptogenesis and epilepsy, and have tried to elucidate the precise mechanisms (Li and Stys, 2001; Chu et al., 2009; Clapcote et al., 2009), although most of which were theoretical speculation. To determine whether significant reductions in Na^+^/K^+^-ATPase in the paramicrogyral cortex can contribute to epileptogenesis, Chu et al. (2009) studied the expression of the α3 isoform of Na^+^/K^+^-ATPase in the freezing lesion (FL) microgyrus model of developing epileptogenesis. Both spatial and temporal altered protein expressions including *ATP1A3* in the cortex have been linked to hyperexcitability, which is a key factor in the pathophysiology of seizure activities. In addition, the functional importance of the *ATP1A3* in regulating epileptiform activity and seizure behavior was shown in a mouse mutagenesis screen, where the *ATP1A3* mutation was found to be associated with seizures (Clapcote et al., 2009). Although previous research has linked *ATP1A3* mutations to the most severe form of infantile epileptic encephalopathy, however, the precise affected brain area of ATP1A3-related epilepsy currently remain unknown. To address this issue, when comparing *ATP*1*A*3^−/−^ and wild-type (*ATP*1*A*3^+/+^) littermates, Ikeda et al. (2017) discovered a considerably increased c-Fos-expressing cells number in several brain regions, with distinct distribution in the cerebellum. Other distribution areas that were strongly positive for *ATP1A3* also include Purkinje cell progenitors in the simple lobule and hemisphere and medial cerebellar nuclei. Brains of *ATP*1*A*3^−/−^ mice have been compared to those of *ATP*1*A*3^+/+^ mice, and it has been discovered that the *ATP*1*A*3^−/−^ brains had greater levels of monoamine neurotransmitters, particularly dopamine and noradrenaline. These findings point to an essential function for *ATP1A3* in the developing and newborn brains. Furthermore, they have shown that ATP1A3^−/−^ consistently has elevated levels of noradrenaline (NA), dopamine (DA), 5-hydroxytryptamine (5-HT), and their metabolites, suggesting that the propulsion afforded by the α3 subunit is essential for monoamine signaling. Drugs that control monoamine signaling may be effective in treating *ATP1A3* mutation-related neurological diseases, where it has been hypothesized that high monoamine levels are a consequence of seizure activity rather than a cause (Ikeda et al., 2017).

**Figure 2:**
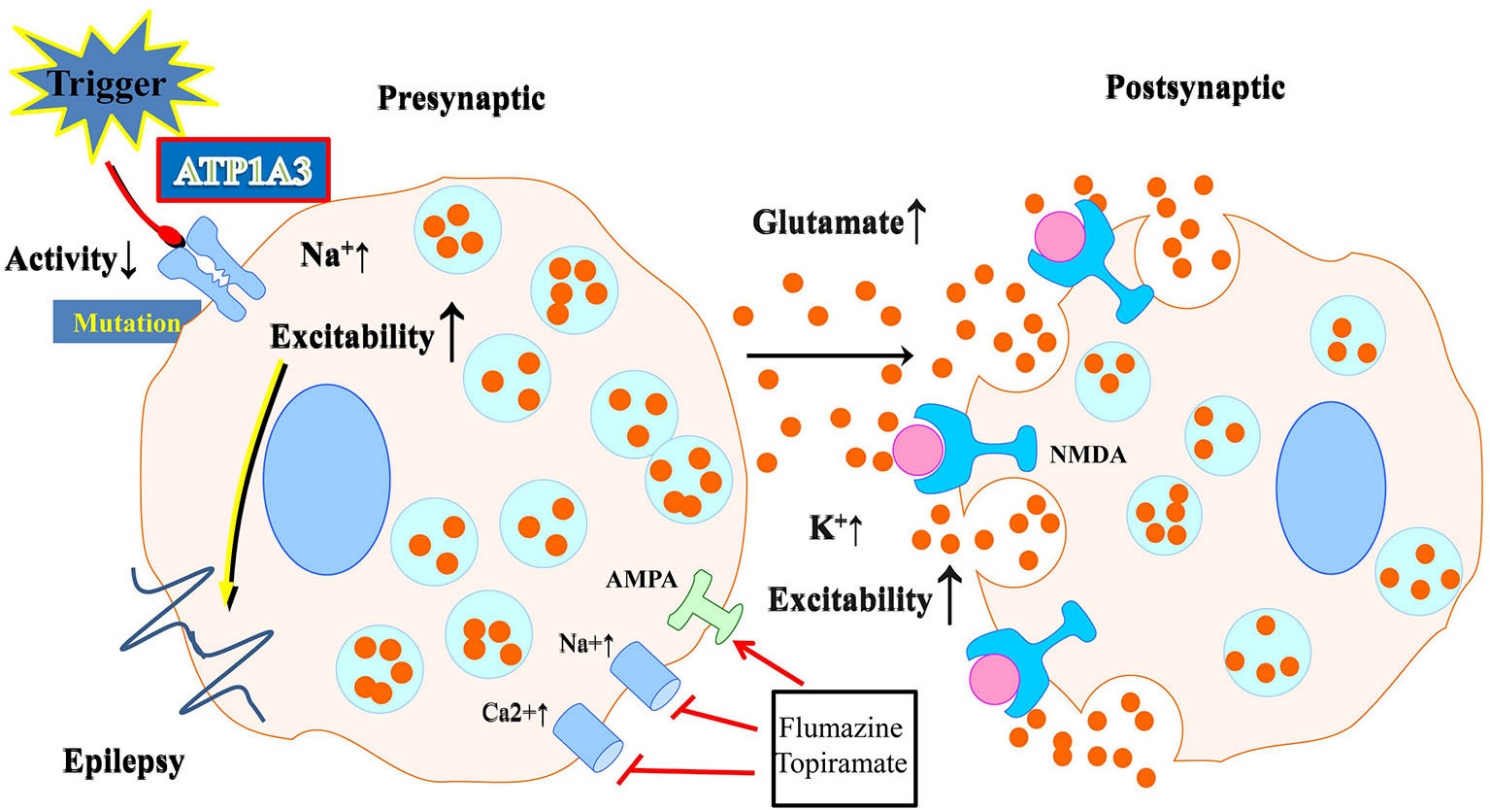
Schematic illustration of the effect of neuronal ATP1A3 mutations on glutamatergic system activity. Abnormalities in Na^+^/K^+^-ATPase may result from mutations. Neurons with an ATP1A3 mutation have increased cellular excitability due to decreased Na^+^/K^+^-ATPase activity and increased intracellular Na^+^. Flunarizine and topiramate could be used as a treatment in some relevant disease cases.

The expression of *ATP1A3* is restricted in neurons. These neurons are especially abundant in the cerebellum and basal ganglia, two brain regions responsible for regulating motor activity, memory, and spatial learning. The *ATP1A3* mutation in neurons, as shown by Kinoshita et al. (2016), reduces the Na^+^/K^+^-ATPase activity and increases intracellular Na^+^, which raises cellular excitability and, thus, affects neuronal activities (Figure 2). Indeed, previous studies have revealed that depolarization, enhanced action potential firing, and further glutamate release may follow from a decrease in the α3 subunit in excitatory neurons (Li and Stys, 2001). Neurotransmitter release may also be affected by a lack of α3 at presynaptic terminals. The impact of *ATP1A3* mutation on epilepsy prevalence may partly be mediated *via* these mechanisms. Treatment options may include flunarizine and topiramate. Flunarizine is a Ca^+^ and Na^+^ channel blocker and was used to treat migraine (Kinoshita et al., 2016). Similarly, the mechanism and efficacy of the action of topiramate is unknown and may include inhibition of 2-amino-3-(3-hydroxyl-5-methyl-4-isoxazolyl) propionic acid (AMPA) receptors as well the carbonic anhydrase, thereby lowering extracellular pH (Hoei-Hansen et al., 2014; Yang et al., 2014). Both of them could be of great potential to be treatment options.

Although substantial hints lead researchers to make such theoretical speculations that *ATP1A3* mutation impacts the normal ion exchange and homeostasis within and outside the neuronal membrane, leading to hyperexcitation and following the occurrence of epilepsy, more evidence obtained from multifaceted experiments, including *in vitro/in vivo* electrophysiology and genetical interferences, are still needed. On the contrary, deficits in glutamatergic control may contribute to various CNS disorders brought on by age-related and mutagenic changes in Na^+^/K^+^-ATPase function. Changes in brain metabolism have been associated with many poorly controlled neurodegenerative disorders including epilepsy. Research into Na^+^/K^+^-ATPase, including how it is regulated by the environment, epigenome, and genome, is expected to be useful in developing novel pharmacological and non-pharmaceutical therapies because of its key role in improving synaptic functioning and energy control (Kinoshita et al., 2016).

### 3.2. Epilepsy animal models based on genetic interference of ATP1A3

Given that disrupting *ATP1A3* functions would directly induce neuronal hyperexcitability and result in seizures, it provides an alternative way to reproduce epileptic animals through genetic modification of *ATP1A3*. Previously, Clapcote et al. described an epileptic mouse model in which a mutation in *ATP1A3* caused seizures (Clapcote et al., 2009). Through *in vivo* mutagenesis with N-nitroso-N-ethylurea (ENU), they were able to generate a mutant termed *Myshkin* (*Myk*), which displays convulsions and neuronal hyperexcitability in heterozygotes (*Myk*^−/+^) and neonatal death in homozygotes (*Myk*/*Myk*). Complex partial and secondary generalized seizures in these mice are caused by a single mutation in the *ATP1A3* gene (I810N in exon 18), which is unique to the Myshkin allele and accounts for all of the diseases. They found that *ATP1A3* plays a crucial role in regulating epileptiform activity and seizure behavior. Seizures (spontaneous and those triggered by vestibular stress), medial temporal sclerosis, sleep disturbances, and various cognitive, motor, social, and behavioral impairments are presented in *Myk*^−/+^ mice (Clapcote et al., 2009; Kirshenbaum et al., 2016). Consistent with the involvement of ATP1A3 in the fast extrusion of Na^+^ following high neuronal activity, the hippocampal CA3-CA1 pathway becomes hyperexcitable in hippocampal slices from *Myk*^−/+^ mice when subjected to high-frequency synaptic activity, such as that induced by stress (Clapcote et al., 2009; Azarias et al., 2013). In addition, when exposed to vestibular stress, *Myk*^−/+^ mice backcrossed 20 generations to C57BL/6NCr (N20) exhibited a considerably reduced drop in adult brain Na^+^/K^+^-ATPase activity and did not demonstrate seizure activity in electrocorticography recordings, in contrast to N12 C57BL/6NCr animals (Kirshenbaum et al., 2011). Two crosses (N2) to the seizure-prone FVB/NCr strain (Goelz et al., 1998) resulted in significant levels of focal reactive astrogliosis and microglial activation in the hippocampus. In contrast, these changes were not seen in N12 C57BL/6NCr *Myk*^−/+^ mice, providing further evidence for the notion that genetic background can influence phenotype (Clapcote et al., 2009). Generally speaking, these mutation-related phenotypes could provide more optimized animal models for exploring the roles of *ATP1A3* in epilepsy, and furthermore, could provide a useful platform for screening effective treatments of certain epileptic phenotypes.

## 4. Conclusion and future prospections

Undoubtedly, Na^+^/K^+^-ATPase function is closely related to the emergence of epileptiform activity, due to its crucial role in maintaining normal ion homeostasis. As this review introduces, mutations in *ATP1A3*, which is a crucial gene encoding the key α subunit, somehow contribute to epileptogenesis and concomitant seizure activities. In this review, we focused on the role of *ATP1A3* alterations in epilepsy, followed by potential mechanisms, by summarizing both clinical and laboratory findings. Our current review highlights that inactivating mutation of *ATP1A3* is a plausible mechanism for human epilepsy due to the high degree of similarity between the various Na^+^/K^+^-ATPase isoforms of mice and humans. Based on current research, more efforts should be directed toward launching more large-scale clinical research to further explore *ATP1A3* alteration significance in the incidence of epilepsy, disease severity, or other specific clinical characteristics. In addition, more laboratory efforts were also needed to in-depth clarify the precise mechanisms, mainly the close association between *ATP1A3* dysfunction and the genesis of epileptic excitatory circuits. It could be proposed that inhibiting ATP1A3 mutations might be a potential treatment for severe epilepsy, and more efforts should be directed toward clarifying the side effects of certain strategies *via* impeding ATP1A3 mutations. In addition, systematic research regarding the ATP1A3 gene mutations in different populations could be of great significance and need to be further explored in the near future. Epilepsy is currently demonstrated to be one consequence of ATP1A3 alteration; however, whether epilepsy could be also a cause of ATP1A3 alteration still need more research. Finally, the most anticipated research of great significance would be seeking potential therapeutic strategies targeting *ATP1A3* in near future.

## Author contributions

SZ and Y-LL wrote the manuscript. YG, CX, and ZC contributed to the revision of the manuscript. All authors contributed to the article and approved the submitted version.
